# Expansion of circulating TFH cells and their associated molecules: involvement in the immune landscape in patients with chronic HBV infection

**DOI:** 10.1186/1743-422X-11-54

**Published:** 2014-03-21

**Authors:** Ting-Ting Hu, Xiao-Fei Song, Yu Lei, Huai-Dong Hu, Hong Ren, Peng Hu

**Affiliations:** 1 Department of Infectious Diseases, Institute for Viral Hepatitis, Key Laboratory of Molecular Biology for Infectious Diseases, Ministry of Education, Second Affiliated Hospital of Chongqing Medical University, 74# Linjiang Road, Yuzhong District, Chongqing 400010, China

**Keywords:** T follicular helper cells, Hepatitis B virus, Interleukin 21, Inducible costimulator

## Abstract

**Background:**

Blood CXCR5+CD4+ T cells are defined as circulating T follicular helper (TFH) cells, which is required for effective humoral immunity. This study aimed to investigate the role of circulating TFH cells in patients with chronic hepatitis B virus (CHB) infection.

**Methods:**

The frequency and phenotype of circulating TFH cells were monitored by flow cytometry in CHB patients and in healthy controls (HC). The expression of BCL-6, IL-21, IL-4, CXCR5, and IL-6R mRNA was analyzed using real-time PCR. Serum HBsAg, HBeAg, HBeAb, HBV DNA loads, ALT and AST were determined. The potential association of the frequency of TFH cells and their surface markers with clinical parameters was assessed.

**Results:**

The frequency of CXCR5+CD4+ T cells was increased in CHB patients and positively correlated with ALT and AST but not with HBV DNA loads. Moreover, an expansion of ICOS-, PD-1-, CD40L-, and IL-21R-expressing TFH cells occurred in CHB patients, but failed to correlate with ALT, AST and HBV DNA loads. Interestingly, the frequency of CXCR5+CD4+ T cells and ICOS+CXCR5+CD4+ T cells was significantly higher in HBeAg positive CHB patients than in HC. Additionally, the percentages of CXCR5+CD4+ T cells were positively correlated with AST, and ICOS-expressing CXCR5+CD4+ T cells were negatively correlated with HBV DNA loads. No significant differences in the frequency of CXCR5+CD4+ T cells were observed between inactive carrier (IC) patients and healthy controls. However, ICOS-, PD-1-, CD40L-expressing TFH cells were increased in IC patients and positively correlated with AST. Furthermore, the expression of BCL-6, IL-21, IL-4, CXCR5, and IL-6R mRNA in TFH cells was higher in CHB patients than in HC.

**Conclusions:**

These data demonstrate that circulating TFH cells may participate in HBV-related immune responses. In addition to the frequency of TFH cells, the phenotype of these cells plays an important role in CHB patients.

## Introduction

Approximately 350 million people worldwide are carriers of hepatitis B virus (HBV), and half a million to 1 million of these carriers die from liver diseases each year [1]. Therapeutic management of chronic hepatitis B patients can stop or slow the progression of the disease and reduce complications, but it is impossible to eradicate HBV and reverse liver damage [1,2]. The outcome of infection and the pathogenesis of liver disease are determined by dynamic interactions between the virus and the host immune system [3]. Thus, elucidating the immune mechanisms that underlie these interactions is critical. Previous studies suggested that the innate and cellular immune responses are major contributors to HBV clearance [4]. However, the role of humoral immunity in controlling HBV remains poorly characterized.

Antibody production by B cells is critical for the clearance of pathogens and for the establishment of long-term humoral immunity [5]. T follicular helper (TFH) cells are the major CD4^+^ T cell subset that is essential for full B cell responses, including germinal center reactions, isotype class switching, and antibody affinity maturation [6]. No unique markers of TFH cells have been reported, and TFH cells are currently defined using a combination of several markers that are directly related to the functions of these cells [6]. TFH cells express large amounts of B cell lymphoma 6 (Bcl-6) [7], which is necessary and sufficient for the development of TFH cells in vivo [8]. Chemokine (C-X-C) receptor 5 (CXCR5) is transiently expressed on CD4^+^ T cells during their interaction with peptide-MHC, but TFH cells can be distinguished from other T cells by their sustained expression of high levels of CXCR5 [9]. CXCR5 promotes the colocalization of T and B cells [10]. TFH cells express surface molecules that play functional roles in T cell and B cell collaboration, such as inducible costimulator (ICOS), programmed cell-death protein 1 (PD-1), and CD40 ligand (CD40L). TFH cells also secrete interleukin 4 (IL-4) and interleukin 21 (IL-21), which are cytokines that promote growth, differentiation, and class switching in B cells [11].

The unique localization of TFH cells within germinal centers is a fundamental feature of this subset of T cells [12].However, it is difficult to obtain lymphoid tissue from patients for research purposes. Therefore, a surrogate strategy is required to assess the quality of TFH cell responses in humans. CXCR5^+^CD4^+^ T cells in the blood may serve as counterparts of TFH cells for this purpose. CXCR5^+^CD4^+^ T cells are known as circulating TFH cells; these cells share functional properties with TFH cells and appear to represent the circulating memory compartment of TFH cells [13-15]. In addition, several studies demonstrated that CXCR5^+^CD4^+^ T cells derived from both the circulation and germinal centers potently induce antibody production during co-culture with B cells in vitro [14,16,17]. The role of blood CXCR5^+^CD4^+^T cells in patients with autoimmunity has been explored [13,14], but little is known about the role of circulating CXCR5^+^CD4^+^ T cells in patients with chronic HBV infection. In this study, we explored the frequency and phenotype of circulating TFH cells in patients with chronic HBV infection and examined the potential association of CXCR5^+^CD4^+^ T cells with clinical parameters. This study may provide novel insights regarding the role of circulating CXCR5^+^CD4^+^ T cells in patients with chronic HBV infection.

## Results

### Clinical data from subjects in the five groups

Table 1 presents the demographic and clinical characteristics of subjects from the chronic HBV infection and HC groups. A total of 38 patients with chronic HBV infection were recruited, including 17 males and 21 females with ages ranging from 7 to 60 years. The subjects were divided into the following groups according to AASLD guidelines [18]: HBeAg-positive chronic hepatitis B (HBeAg + CHB) (n = 14); immune tolerant carrier (IT) (n = 8); inactive carrier (IC) (n = 10); and HBeAg-negative chronic hepatitis B (HBeAg- CHB) (n = 6). In addition, 12males and 13 females with ages ranging from 23 to 64 years were included as controls. The patients with chronic HBV infection were comparable to the healthy controls with respect to sex and age. All patients with chronic HBV infection were positive for HBV DNA, and half of these patients had elevated levels of ALT and/or AST. Notably, the healthy controls were negative for HBV DNA and had normal ALT and AST levels.

**Table 1:** The demographic and clinical characteristics of subjects

**Parameters**	**HC**	**CHB (HBeAg+)**	**IT**	**IC**	**CHB (HBeAg-)**
Number	25	14	8	10	6
Age (years)					
Mean ± SD	30.6 ± 10.6	27.9 ± 12.0	26.8 ± 6.7	46.1 ± 14.8	42.8 ± 12.2
Sex N (%)					
Male	12(48.0)	5(35.7)	5(62.5)	5(50.0)	2(33.3)
Female	13(52.0)	9(64.3)	3(37.5)	5(50.0)	4(66.7)
HBV-DNA (log_10_ copies/mL)					
Mean ± SD	NA	6.5 ± 0.4	7.2 ± 0.8	2.0 ± 1.3	4.6 ± 0.2
ALT (U/L)					
Mean ± SD	≤40	504.1 ± 635.3	26.1 ± 8.4	21.7 ± 9.9	118.5 ± 56.7
AST (U/L)					
Mean ± SD	≤40	430.3 ± 657.6	23.6 ± 6.5	27.8 ± 9.3	58.3 ± 21.4
HBeAg/Anti-HBe	0/0	14/0	8/0	0/10	0/6

NA, not applicable; HC, healthy control; CHB(HBeAg+), HBeAg-positive chronic hepatitis B; IT, immune tolerant carrier; IC, inactive carrier; CHB(HBeAg-), HBeAg-negative chronic hepatitis B.

### Significantly increased frequency of CXCR5^+^CD4^+^ T cells in patients with chronic HBV infection compared to healthy controls

**Figure 1:**
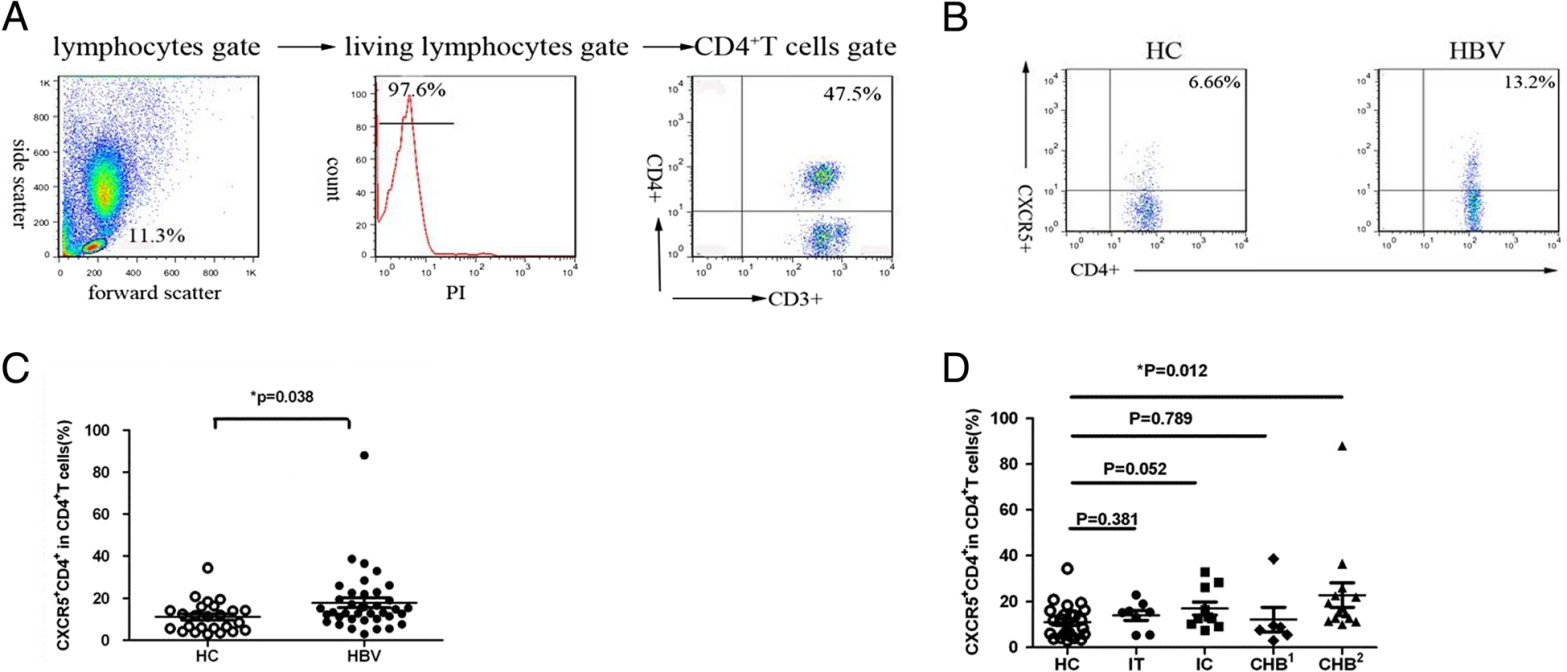
**Fluorescence-activated cell sorter (FACS) analysis of the frequency of peripheral blood CXCR5**^**+**^**CD4**^**+ **^**T cells in patients with chronic HBV infection patients. (A)** Gating strategy. **(B)** The percentage of circulating CXCR5^+^CD4^+^ T cells was compared between the two groups. **(C)** The percentage of CXCR5^+^CD4^+^ T cells was significantly higher in patients with chronic HBV infection than in healthy controls (P < 0.05). **(D)** Comparison of the frequency of CXCR5^+^CD4^+^ T cells in the IT, IC, CHB(HBeAg+), CHB(HBeAg-) and HC groups. HC, healthy controls; HBV, patients with chronic HBV infection; IT, immune tolerant carrier; IC, inactive carrier; CHB^1^, HBeAg-negative chronic hepatitis B; CHB^2^, HBeAg-positive chronic hepatitis B; PI, propidium iodide. The horizontal lines represent the median values (±SEM).

To determine the potential role of peripheral CXCR5^+^CD4^+^ T cells in patients with chronic HBV infection, the frequency of peripheral blood CXCR5^+^CD4^+^ T cells within the CD4^+^ T cell population was monitored by flow cytometry (Figure 1). The percentages of CXCR5^+^CD4^+^ T cells were 17.87% (2.95-88.03%) in the chronic HBV infection group and 11.13% (2.92-34.48%) in the HC group. As shown in Figure 1C, the frequency of CXCR5^+^CD4^+^ T cells was significantly higher in patients with chronic HBV infection than in the HC group (p < 0.05). To further characterize the frequency of CXCR5^+^CD4^+^ T cells in patients with chronic HBV infection, it was necessary to determine whether differences existed between the chronic HBV infection subgroup and the HC group. Interestingly, the percentages of CXCR5^+^CD4^+^ T cells were greater in the HBeAg - positive chronic hepatitis B group than in the HC group (p < 0.05) (Figure 1D). In contrast, the frequency of CXCR5^+^CD4^+^ T cells in IT, IC or HBeAg- CHB patients was not significantly different from that observed in the healthy controls (p > 0.05) (Figure 1D). Moreover, no significant differences in the proportions of CXCR5^+^CD4^+^ T cells were observed among the chronic HBV infection subgroups (data not shown).

### Increased expression of ICOS, PD-1, CD40L and IL-21R in CXCR5^+^CD4^+^ T cells in patients with chronic HBV infection

**Figure 2:**
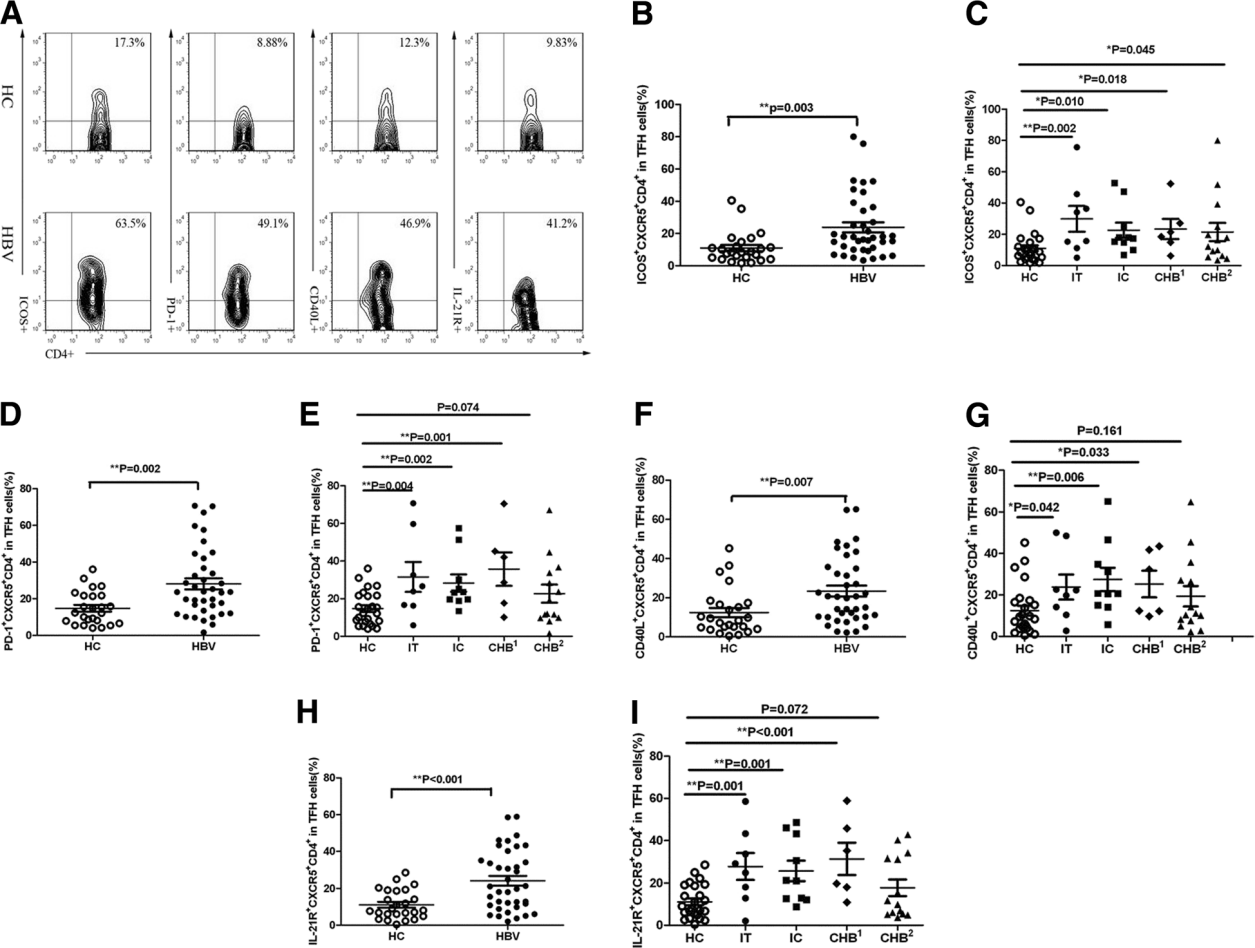
**The phenotype of circulating TFH cells was assessed in patients with chronic HBV infection and in HC. (A)** The expression of ICOS, PD-1, CD40L and IL-21R by CXCR5^+^CD4^+^ T cells were detected by flow cytometry. At least 50,000 events were analyzed for each sample, and the data represent different groups of samples from at least two independent experiments. **(B)**, **(D)**, **(F)** and **(H)** Comparison of the percentages of ICOS^+^CXCR5^+^CD4^+^ T cells, PD-1^+^CXCR5^+^CD4^+^ T cells, CD40L^+^CXCR5^+^CD4^+^ T cells, and IL-21R^+^CXCR5^+^CD4^+^ T cells in the chronic HBV infection and HC groups. **(C)**, **(E)**, **(G)** and **(I)** Comparison of the percentages of ICOS^+^CXCR5^+^CD4^+^ T cells, PD-1^+^CXCR5^+^CD4^+^ T cells, CD40L^+^CXCR5^+^CD4^+^ T cells, and IL-21R^+^CXCR5^+^CD4^+^ T cells between the IT, IC, CHB(HBeAg+), CHB(HBeAg-) and HC groups. CHB^1^, HBeAg-negative chronic hepatitis B; CHB^2^, HBeAg-positive chronic hepatitis B. Each data point represents an individual subject. *, p < 0.05; **, p < 0.01.

A significantly higher frequency of CXCR5^+^CD4^+^ T cells was observed in the chronic HBV infection group than in the HC group. Thus, it was important to determine whether the expression of markers that are typically associated with TFH cells were altered. TFH cells are distinguished from other cell types by the expression of ICOS, PD-1, CD40L, IL-21R, CXCR5, and IL-21. The phenotype of TFH cells was assessed in this study (Figure 2A). Notably, the expression of ICOS was increased in CXCR5^+^CD4^+^ T cells in patients with chronic HBV infection compared to healthy controls (p < 0.05) (Figure 2B). More importantly, the percentages of ICOS^+^CXCR5^+^CD4^+^ T cells were significantly higher in the HBeAg + CHB, IT, IC and HBeAg- CHB groups compared to the HC group (p < 0.05) (Figure 2C). Likewise, differential expression of PD-1 was observed between the patients with chronic HBV infection and the healthy subjects (p < 0.01) (Figure 2D). In addition, the proportions of CD40L^+^CXCR5^+^CD4^+^ T cells and IL-21R^+^ CXCR5^+^CD4^+^ T cells were higher in patients with chronic HBV infection than in the healthy controls (p < 0.01) (Figure 2F, H). Remarkably, significantly higher levels of PD-1^+^CXCR5^+^CD4^+^ T cells, CD40L^+^CXCR5^+^CD4^+^ T cells, and IL-21R^+^ CXCR5^+^CD4^+^ T cells were observed in the IT, IC and HBeAg- CHB groups than in the HC group (p < 0.05) (Figure 2E, G, I). However, no significant difference in the proportions of PD-1^+^CXCR5^+^CD4^+^ T cells, CD40L^+^CXCR5^+^CD4^+^ T cells, and IL-21R^+^ CXCR5^+^CD4^+^ T cells were observed between the HBeAg + CHB group and the HC group (p > 0.05) (Figure 2E, G, I). Further analysis of the chronic HBV infection subgroups revealed no differences between the subgroups (data not shown).

### Patients with chronic HBV infection exhibited overexpression of BCL-6, CXCR5, IL-6R, IL-4, and IL-21 mRNA in CXCR5^+^CD4^+^ T cells

**Figure 3:**
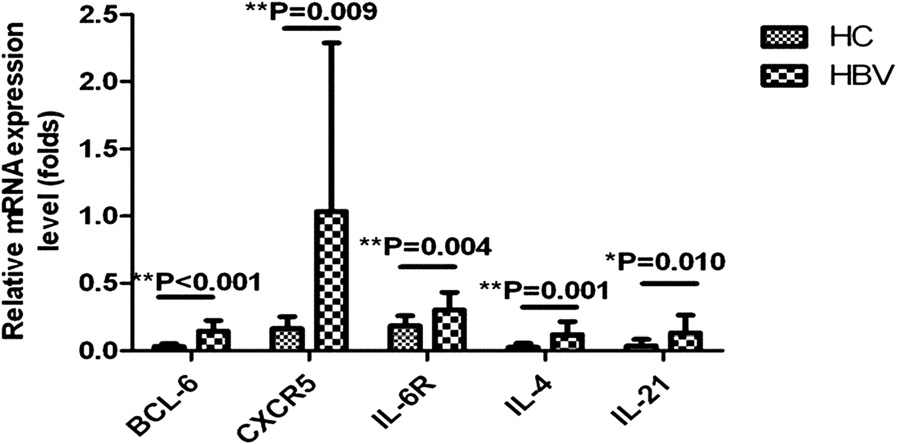
**mRNA expression levels were determined by real-time PCR.** Increased expression of BCL-6, CXCR5, IL-6R, IL-4, and IL-21 mRNA in CXCR5^+^CD4^+^ T cells in patients with chronic HBV infection (n = 16) compared to healthy controls (n = 16). Data are presented as the mean ± SD. *,p < 0.05; **, p < 0.01.

As previously discussed, the two groups exhibited differential expression of ICOS, PD-1, CD40L, and IL-21R. Elucidating the transcription factors, cytokines and additional markers that are associated with TFH cells is critical for an improved understanding of the role of TFH cells. TFH cells rely on the expression of the master regulator transcription factor Bcl-6. CXCR5^+^CD4^+^ T cells in patients with chronic HBV infection significantly up-regulated expression of Bcl-6 mRNA compared to healthy controls (p < 0.01). IL-21 is a typical cytokine for TFH cells; as expected, IL-21 expression was significantly different between the chronic HBV infection and HC groups (p < 0.05). Moreover, the mRNA expression of additional markers that are associated with TFH cells was analyzed using real-time PCR. The expression of IL-6R, IL-4 and CXCR5 mRNA in circulating CXCR5^+^CD4^+^ T cells was higher in patients with chronic HBV infection than in healthy controls (p < 0.05) (Figure 3).

### Correlation of CXCR5^+^CD4^+^ T cells with clinical parameters in patients with chronic HBV infection

**Figure 4:**
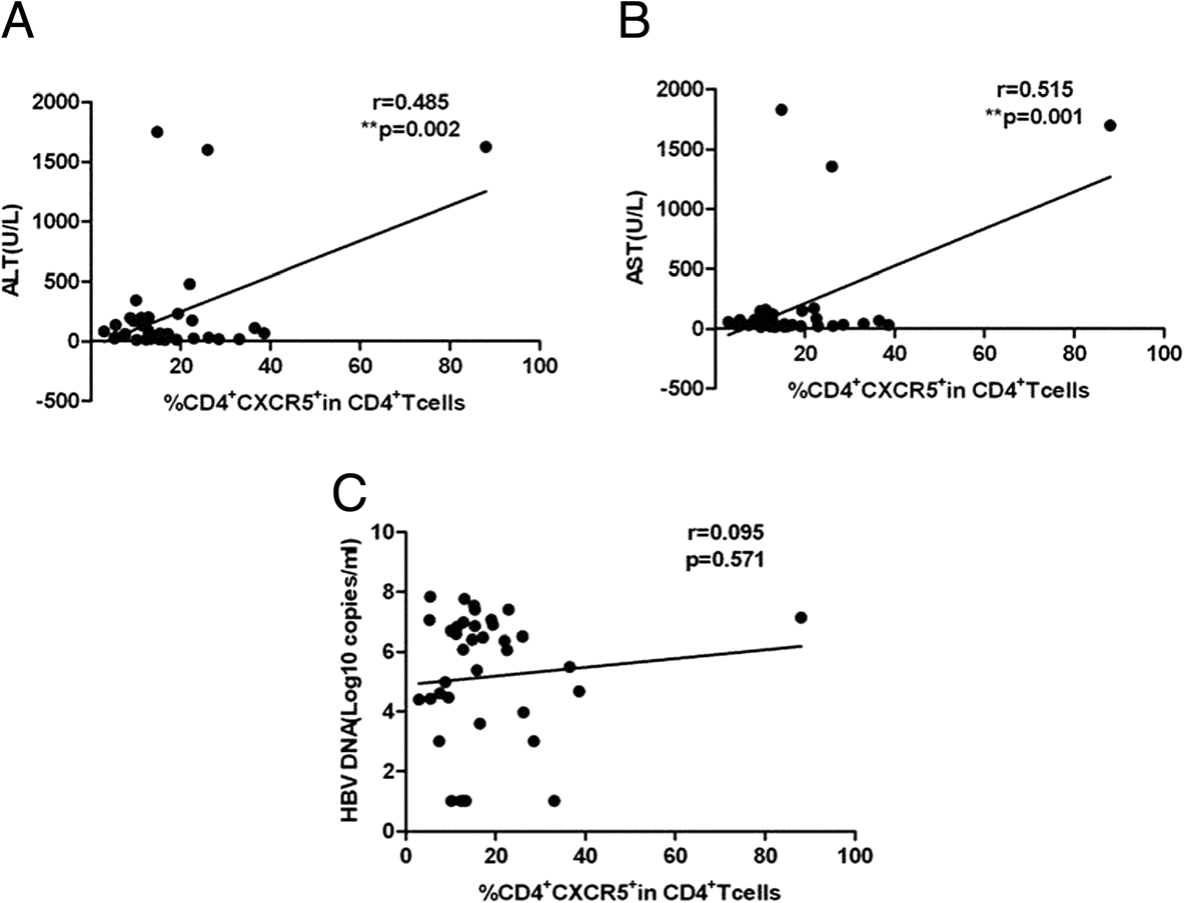
**The correlation between CXCR5**^**+**^**CD4**^**+ **^**T cells and clinical parameters in patients with chronic HBV infection. (A)** The correlation between the percentage of CXCR5^+^CD4^+^ T cells and serum levels of ALT. **(B)** The correlation between the percentage of CXCR5^+^CD4^+^ T cells and serum levels of AST. **(C)** The correlation between the percentage of CXCR5^+^CD4^+^ T cells and HBV DNA loads. Data were expressed as the mean values of individual patients (n = 38) from three separate experiments.

**Figure 5:**
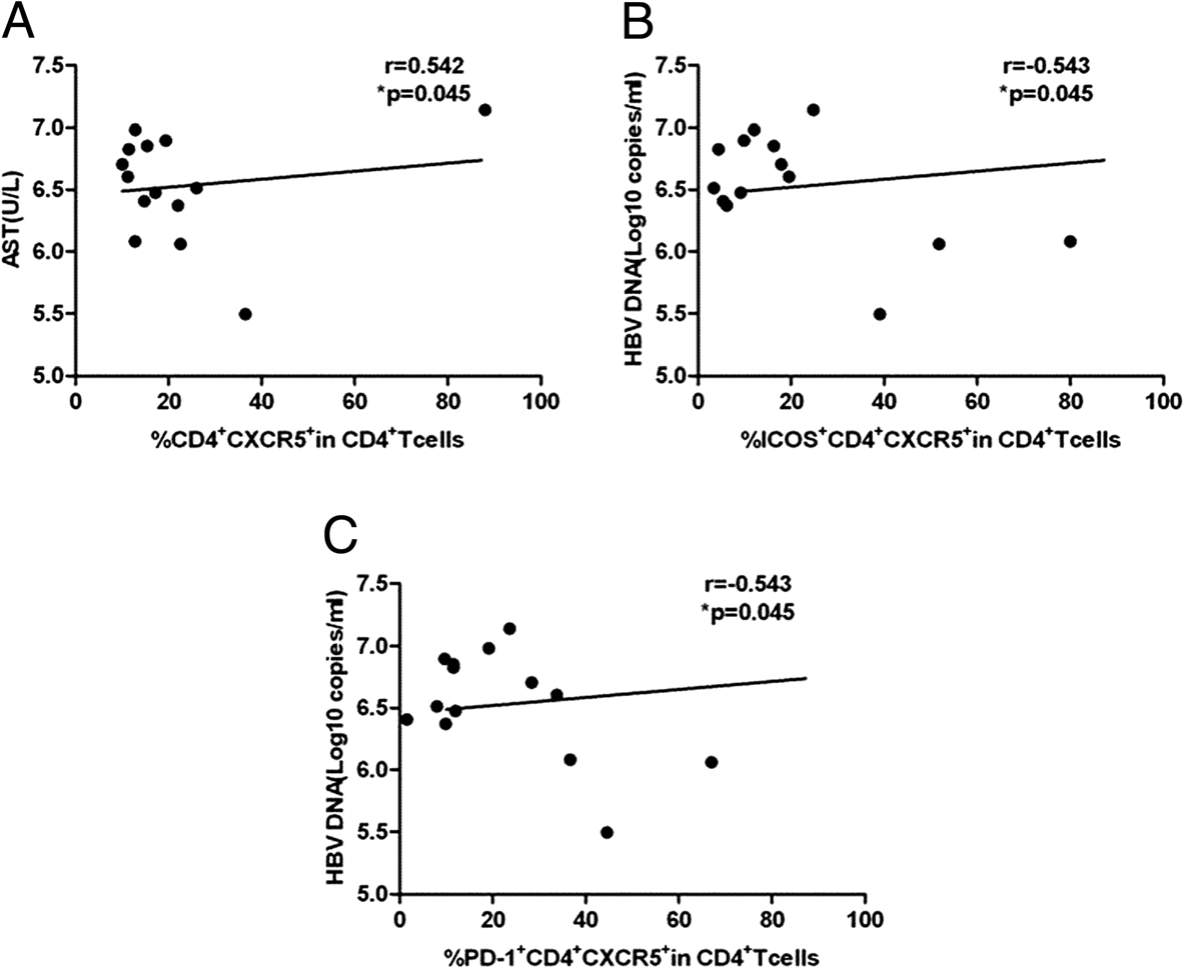
**The correlation between CXCR5**^**+**^**CD4**^**+ **^**T cells and clinical parameters in HBeAg-positive chronic hepatitis B patients. (A)** The correlation between the percentage of CXCR5^+^CD4^+^ T cells and serum levels of AST. **(B)** The correlation between the percentage of ICOS^+^CXCR5^+^CD4^+^ T cells and HBV DNA loads. **(C)** The correlation between the percentage of PD-1^+^CXCR5^+^CD4^+^ T cells and HBV DNA loads. Data were expressed as the mean values of individual patients (n = 14) from three separate experiments.

**Figure 6:**
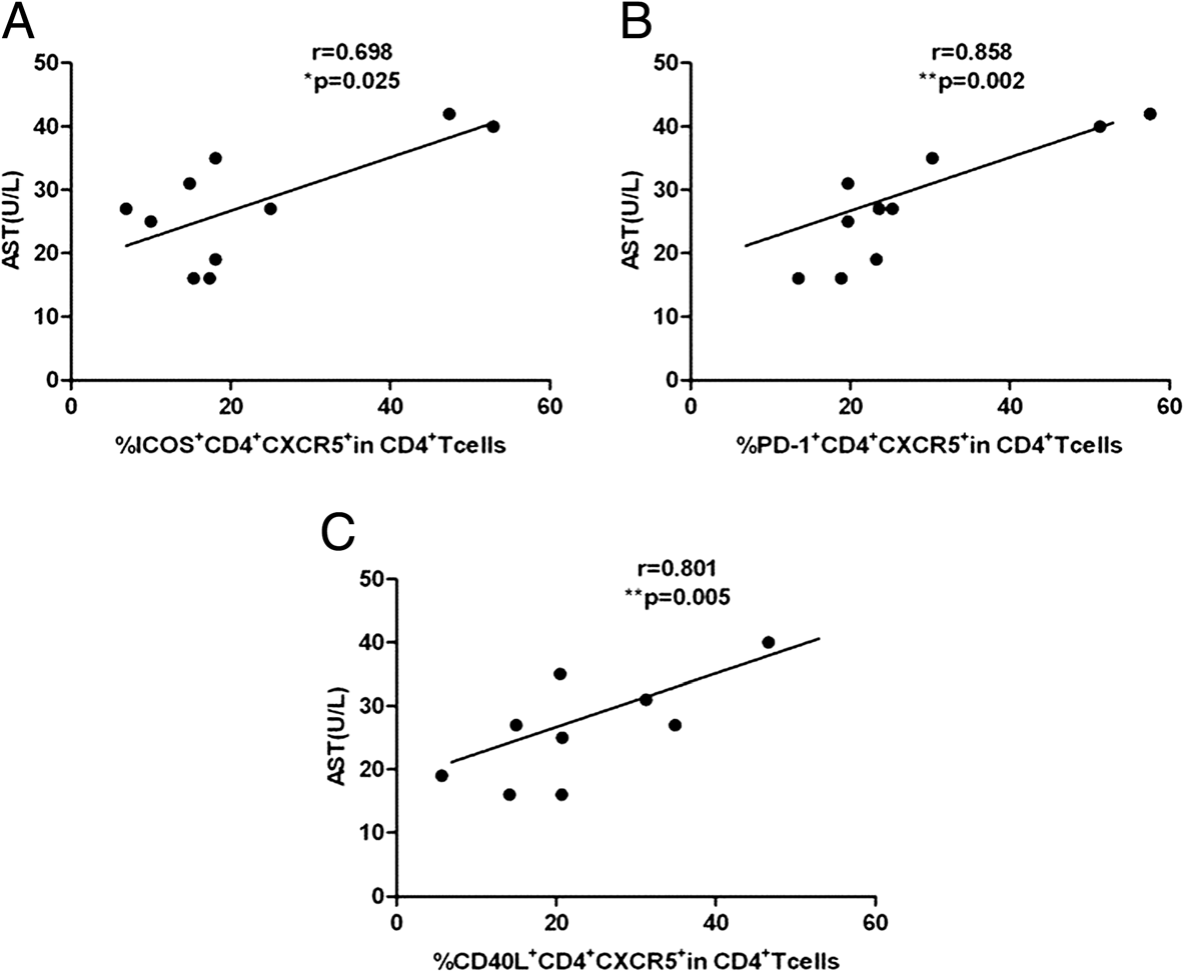
**The correlation between CXCR5**^**+**^**CD4**^**+ **^**T cells and clinical parameters in inactive carriers. (A)** The correlation between the percentage of ICOS^+^CXCR5^+^CD4^+^ T cells and AST. **(B)** The correlation between the percentage of PD-1^+^CXCR5^+^CD4^+^ T cells and AST. **(C)** The correlation between the percentage of CD40L^+^CXCR5^+^CD4^+^ T cells and AST. Data were expressed as the mean values of individual patients (n = 10) from three separate experiments.

Because of the high frequency of TFH cells and the upregulation of a number of molecules associated with TFH cells in patients with chronic HBV infection, we evaluated whether TFH cells were linked to clinical parameters. ALT is a commonly used clinical parameter that is associated with the inflammatory response in patients with chronic HBV infection, and AST is screened to determine the grade of liver injury. The percentage of CXCR5^+^CD4^+^ T cells positively correlated with the levels of serum ALT and AST in patients with chronic HBV infection (r = 0.485, p < 0.01 and r = 0.515, p < 0.01, respectively) (Figure 4A, B). However, no significant correlation was found between the percentages of ICOS^+^CXCR5^+^CD4^+^ T cells, PD-1^+^CXCR5^+^CD4^+^ T cells, CD40 L^+^CXCR5^+^CD4^+^ T cells, or IL-21R^+^CXCR5^+^CD4^+^ T cells and the levels of serum ALT and AST (data not shown). Additionally, the frequencies of CXCR5^+^CD4^+^ T cells, ICOS^+^CXCR5^+^CD4^+^ T cells, PD-1^+^CXCR5^+^CD4^+^ T cells, CD40L^+^CXCR5^+^CD4^+^ T cells, and IL-21R^+^CXCR5^+^CD4^+^ T cells were not correlated with HBV DNA loads (data not shown). Surprisingly, the percentage of CXCR5^+^CD4^+^ T cells in HBeAg + CHB patients was positively correlated with AST (r = 0.542, p < 0.05) (Figure 5A) but not with ALT or HBV DNA levels (data not shown). Moreover, the frequencies of ICOS^+^CXCR5^+^CD4^+^ T cells and PD-1^+^CXCR5^+^CD4^+^ T cells in HBeAg + CHB patients were negatively correlated with HBV DNA loads (r = -0.543, p < 0.05 and r = -0.543, p < 0.05, respectively (Figure 5B, C). Further, the percentage of CXCR5^+^CD4^+^ T cells in IC patients was not correlated with ALT, AST or HBV DNA loads (data not shown). In contrast, the proportions of ICOS^+^CXCR5^+^CD4^+^ T cells, PD-1^+^CXCR5^+^CD4^+^ T cells, and CD40L^+^CXCR5^+^CD4^+^ T cells in IC patients were positively correlated with AST (r = 0.698, p < 0.05; r = 0.858, p < 0.01; and r = 0.801, p < 0.01, respectively) (Figure 6A, B, C).

## Discussion

The role of TFH cells in infectious and autoimmune diseases has attracted recent attention. The mechanisms by which TFH cells develop and the mechanisms by which TFH cells or their associated molecules regulate antibody responses in humans remain largely unknown. Several studies demonstrated that the frequency of CXCR5^+^CD4^+^ T cells was not significantly increased in patients with autoimmune diseases, such as SLE [13], autoimmune thyroid disease [19], and juvenile dermatomyositis [14], compared to healthy controls. In contrast, conflicting results have been obtained concerning the existence of a significantly higher frequency of CXCR5^+^CD4^+^ T cells in patients with chronic HBV infection than in the HC group [20]. Consistent with previous studies, our findings demonstrated that the percentages of CXCR5^+^CD4^+^ T cells were increased in patients with chronic HBV infection compared to healthy controls. The data obtained in this study suggest that chronic HBV infection drives CXCR5^+^CD4^+^ T cell differentiation because in addition to the increased frequency of these cells, the phenotype of CXCR5^+^CD4^+^ T cells is altered.

The function of TFH cells is closely related to a combination of markers. In this study, the expression of ICOS was increased in CXCR5^+^CD4^+^ T cells in patients with chronic HBV infection compared to healthy controls. ICOS is not expressed by resting T cells and is rapidly upregulated after TCR stimulation. Previous studies demonstrated that ICOS was required for Bcl6 upregulation and for the initiation of the TFH cell differentiation program [21]. Reduced numbers of TFH cells were observed in the absence of ICOS signals [22]. ICOS delivers costimulatory signals by binding to ICOS ligand (ICOS-L) expressed on antigen presenting cells. In both mice and humans, the interruption of the ICOS-ICOS-L interaction leads to impaired GC reactions, Ab class switching, and affinity maturation [22,23]. Differential expression of PD-1 was also observed between the two groups. PD-1 signaling plays a major role in inhibiting T cell responses. Previous studies demonstrated that PD-1 was elevated during a viral infection to inhibit chronically activated T cells from causing immunopathology or becoming autoreactive [24]. Accumulating evidence indicates that PD-L1- and/or PD-L2-expressing B cells interacted with PD-1^+^ TFH cells to regulate germinal center B cell survival and the formation and affinity of long-lived plasma cells [25]. We also found that the proportions of CD40L^+^CD4^+^CXCR5^+^ and IL-21R^+^CD4^+^CXCR5^+^ cells were higher in patients with chronic HBV infection than in healthy controls. The CD40L/CD40 interaction promotes germinal center formation, immunoglobulin isotype switching, and somatic hypermutation of the Ig to enhance affinity for antigens, as well as the subsequent formation of long-lived plasma cells and memory B cells [26].

BCL-6 was recently identified as a master regulator of TFH differentiation. Constitutive expression of Bcl-6 drives TFH differentiation in vivo [27]. Previous work demonstrated that GC TFH cells expressed a high level of Bcl-6, while blood CXCR5^+^CD4^+^ T cells failed to express elevated levels of the Bcl-6 protein [28]. In contrast to previous studies, blood CXCR5^+^CD4^+^ T cells in patients with chronic HBV infection exhibited significantly up-regulated levels of Bcl-6 mRNA compared to healthy controls. IL-21 has been reported to play a role in TFH cell formation and in B cell growth, survival, and isotype switching [29-32]. Our data demonstrated that IL-21 mRNA was highly expressed in circulating TFH cells in patients with chronic HBV infection. This finding suggests that circulating TFH cells can help naïve B cells to produce antibody via IL-21 in patients with chronic HBV infection. The observation that TFH cells produce IL-4 after parasite infection was confirmed by several studies [33,34]. IL-4 expression by GC TFH cells that lack other Th2 characteristics is important for murine B cell IgG1 class switching.

Our study reveals that circulating TFH cells may be involved in HBV-related immune responses via transcription factors in combination with induction signals and secreted cytokines. We further clarify the relationship between circulating TFH cells and clinical parameters in patients with chronic HBV infection. The percentage of CXCR5^+^CD4^+^ T cells was positively correlated with serum levels of ALT and AST. However, the frequency of CXCR5^+^CD4^+^ T cells was not correlated with HBV DNA loads. These findings indicate that blood CXCR5^+^CD4^+^ T cells are associated with HBV-related liver damage. TFH cells may participate in the inflammatory response, but these cells have no relationship with HBV DNA load. Moreover, no significant correlations were observed between the percentage of ICOS^+^CXCR5^+^CD4^+^, PD-1^+^CXCR5^+^CD4^+^, CD40L^+^CXCR5^+^CD4^+^, or IL-21R^+^CXCR5^+^CD4^+^ T cells and serum levels of ALT, AST or HBV DNA loads. The frequency of CXCR5^+^CD4^+^ T cells was positively correlated with serum levels of ALT and AST, but no significant correlation was found between phenotype and serum levels of ALT and AST. Further studies are needed to determine whether the expression of CXCR5^+^CD4^+^ T cells varies at different stages of the chronic HBV infection process. In this study, the frequency of CXCR5^+^CD4^+^ T cells and ICOS^+^CXCR5^+^CD4^+^ T cells was significantly higher in HBeAg + CHB patients than in HC, and the percentage of CXCR5^+^CD4^+^ T cells in HBeAg + CHB patients was positively correlated with AST. In addition, the proportion of ICOS^+^CXCR5^+^CD4^+^ T cells in HBeAg + CHB patients was negatively correlated with HBV DNA loads. Further, the frequency of ICOS^+^CXCR5^+^CD4^+^ T cells, PD-1^+^CXCR5^+^CD4^+^ T cells, and CD40L^+^CXCR5^+^CD4^+^ T cells in IC patients was positively correlated with AST. Our findings reveal that TFH cells may reflect active immune responses because TFH cells are specialized providers of B cell help and promote humoral responses against viral infection. This positive correlation also suggests that the frequency and phenotype of CXCR5^+^CD4^+^ T cells is associated with HBV-related liver damage.

## Conclusion

Our results demonstrated that the levels of CXCR5^+^CD4^+^ T cells and the expression of ICOS, PD-1, CD40L, and IL-21R in CXCR5^+^CD4^+^ T cells were increased in patients with chronic HBV infection compared to healthy controls. Moreover, CXCR5^+^CD4^+^ T cells were positively correlated with serum levels of ALT and AST in patients with chronic HBV infection, indicating that circulating counterparts of TFH cells may be involved in HBV-related immune responses. Further functional investigations of these cells may elucidate the pathogenesis of chronic HBV infection.

## Materials and methods

### Patients

A total of 38 patients with chronic HBV infection were recruited from the outpatient service of the Second Affiliated Hospital of Chongqing Medical University from July to November 2012, and 25 healthy volunteers were selected as controls. Individual subjects with HBV infection were confirmed to be positive for both HBsAg and detectable HBV virions for more than 6 months. Subjects infected with the hepatitis A, C, D, or E viruses or the human immunodeficiency virus and patients with a history and clinical features of drug-induced liver injury, alcoholic hepatitis, autoimmune diseases and steatohepatitis were excluded. All of the included patients were naïve and had not been previously treated with nucleoside/nucleotide analog antiviral or immunomodulatory drugs. This study conformed to the ethical guidelines of the Declaration of Helsinki and was approved by the Ethics Committee of the Second Affiliated Hospital of Chongqing Medical University. All subjects provided written informed consent.

### Samples

Peripheral blood samples were obtained from individual subjects with written informed consent*.* Levels of HBV DNA were detected using a fully automated real-time PCR machine and the luciferase quantitation detection kit, with a detection limit of 300 copies/mL (Roche Amplicor, Basel, Switzerland). Serum markers of HBV, anti-HAV, anti-HCV and anti-HEV were determined via the Roche electrochemical luminescence method using an Architect i2000 system (Abbott Laboratories, Abbott Park, IL, USA). Serum anti-HDV was analyzed by ELISA according to the manufacturer’s instructions (Kehua Bio-Engineering Co, Ltd, Shanghai, China). Serum levels of AST and ALT were detected using the Automatic Biochemistry Analyzer (Beckman LX-20, Beckman, USA).

### Flow cytometry analysis

Five milliliters of heparinized peripheral venous blood was obtained from either healthy volunteers or patients with chronic HBV infection. After removing plasma, the red blood cells were lysed using an NH_4_Cl lysis solution. Flow cytometry analysis was performed on 10^6^ cells per tube using the following fluorochrome-conjugated antibodies: anti-CD3–phycoerythrin (PE)–cyanine (CY) 7 (eBioscience, San Diego, CA, USA), anti- CD4–fluorescein isothiocyanate (FITC) (BD Company, San Jose, CA, USA), anti- CXCR5–allophycocyanin (APC) (BD Company, San Jose, CA, USA), anti-ICOS–PE (BD Company, San Jose, CA, USA), anti-PD1-PE (BD Company, San Jose, CA, USA), anti-CD40L–PE (eBioscience, San Diego, CA, USA), and anti-IL-21R-PE (BD Company, San Jose, CA, USA). Isotype-matched control antibodies (Beckton Dickinson, San Jose, USA) were used to correct nonspecific binding. After staining for 30 min at 4°C, the cells were washed twice with PBS containing 0.5% bovine serum albumin and subsequently analyzed using a FACS Canto II cytometer and FACSDiva software, version 4.1 (Becton Dickinson).

### MACS cell separation

CXCR5^+^CD4^+^ T cells were isolated from PBMCs using an autoMACS to achieve >95% purity, as previously described. The employed MACS Cell Separation Reagents contained a CD4^+^ T cell biotin–antibody cocktail, a CXCR5-biotin-antibody, and micro-beads. CD4^+^ T cells were negatively selected using a CD4^+^ T cell isolation kit (Miltenyi Biotec, Germany), yielding populations of CD4^+^ cells with 96–99% purity. Next, CXCR5^+^ T cells were separated from CD4^+^ T cells on the AutoMACS via repetitive separation steps using a positive selection kit (Miltenyi Biotec).

### Real-time PCR

Total RNA was extracted from blood TFH cells using Trizol (Invitrogen) according to the manufacturer’s instructions. The concentration and the purity of the RNA were determined by absorbance at 260/280 nm, and cDNA was synthesized using the PrimeScript RT reagent Kit (TaKaRa, Japan). Relative levels of gene expression were measured by real-time PCR using the SYBRGreen master mix reagent in an ABI PRISM 7300 sequence detection system. The following primers were used: GAPDH (NSO_1236141_039, NSO_1236141_040, Invitrogen), BCL-6 (HP 205513, Origene), CXCR5 (HP205521, Origene), IL-6R (HP200535, Origene), IL-4 (HP200556, Origene), and IL-21 (HP214222, Origene). Amplification began with an initial denaturation for 30 seconds at 95°C followed by 40 cycles of denaturation at 95°C for 5 seconds and annealing and extension at 60°C for 31 seconds. The plate was subsequently read. Each sample was tested in triplicate, and the average values were used for subsequent calculations. The expression of each gene was normalized to the housekeeping gene GAPDH, and expressed values relative to control were calculated using the ^△△^CT method. Amplified products were visualized using a UV transilluminator in combination with 1.2% agarose gel electrophoresis and ethidium bromide staining.

### Statistical analysis

All statistical tests were performed using SPSS software, version 17.0 (SPSS Inc., Chicago, IL, USA). Data were presented as the mean ± SD. The t-test was used to compare two independent variables. Correlations between variables were evaluated using the Pearson correlation test. A two-tailed P < 0.05 was considered statistically significant.

## Competing interests

The authors declare that they have no competing interests.

## Authors’ contributions

TTH and XFS performed all experiments and data analysis. TTH created the first draft of the manuscript. YL participated in the design of the study. HDH and HR provided assistance with experimental concepts. PH conceived and designed the study and finished the final manuscript. All authors read and approved the final manuscript.
